# Editorial: Special Issue “Molecular Epidemiology of Antimicrobial Resistance”

**DOI:** 10.3390/microorganisms11030579

**Published:** 2023-02-24

**Authors:** Raffaele Zarrilli, Tommaso Giani, Rémy A. Bonnin

**Affiliations:** 1 Department of Public Health, University of Naples Federico II, 80131 Naples, Italy; 2 Department of Experimental and Clinical Medicine, University of Florence, 50139 Florence, Italy; 3 Team Resist, UMR-1184 (INSERM-Université Paris-Saclay-CEA), LabEx Lermit, Faculty of Medicine, 94270 Le Kremlin-Bicêtre, France

Antimicrobial resistance and multidrug-resistant organisms currently constitute a severe public health problem. Multidrug-resistant organisms are resistant to multiple antibiotic classes, resulting in limited therapeutic options and difficult-to-treat health-care-associated and community infections, with high morbidity and mortality rates. In particular, carbapenem-resistant (CR) *Acinetobacter baumannii*, CR *Pseudomonas aeruginosa*, CR *Enterobacterales,* methicillin-resistant *Staphylococcus aureus* and vancomycin-resistant *Enterococcus* spp. are recognized by the World Health Organization as global priority pathogens of critical or high priority [1].

This Special Issue was dedicated to updates on the “Molecular Epidemiology of Antimicrobial Resistance”. Two manuscripts investigated new patterns in the epidemiology of infections caused by isolates belonging to the *A. baumannii-calcoaceticus* complex. The epidemics of *A. baumannii* are characterized by the spread of multidrug-resistant clonal lineages [2]. Monnheimer et al. [3] analyzed the phenotypic and genotypic features of carbapenem-resistant *A. baumannii* isolates responsible for wound infections in Ghana. Chopjitt et al. [4] investigated the genomic epidemiology of extensively drug-resistant *Acinetobacter pittii* isolates from Taiwan and China.

The molecular determinants, genetic and genomic elements of antimicrobial resistance in *Enterobacterales*, have been the subject of four studies. The research paper by Bilal et al. [5] reported the occurrence of *bla_NDM-1_* bearing IncX3 plasmid in clinically isolated ST11 *Klebsiella pneumoniae* from Pakistan. A new resistance-mediating plasmid chimera was detected in *bla_OXA-48_*-positive *Klebsiella pneumoniae* strain at a German university hospital [6]. HI2 plasmids mobilising the carbapenemase gene *bla_IMP-4_* were identified both in *Escherichia coli* Australian clinical samples and in multiple sublineages of *E. coli* ST216 colonising silver gulls [7]. Edowik et al. [8] demonstrated that the amino acid changes T55A, A273P and R277C in the beta-Lactamase CTX-M-14 render *E. coli* resistant to the antibiotic nitrofurantoin, which is used as first-line treatment of urinary tract infections. Lin et al. [9] showed that the dissemination of multidrug-resistant composite transposons MESPM1 or MES6272 between *Enterococcus* and ST59 *S. aureus* was mediated by insertion sequence IS1216V.

One manuscript focussed on diagnostics of antimicrobial resistances. Vasilakopoulou et al. [10] reported the evaluation of the NG-Test CARBA 5 immunochromatographic assay for detecting KPC, NDM and VIM-producing carbapenemases organisms directly from rectal swabs.

Therapeutics and management for the prevention and control of infections caused by multidrug-resistant organisms were investigated in three manuscripts. Yang et al. [11] analyzed the in vitro and in vivo efficacies of ceftazidime–avibactam and aztreonam–avibactam against carbapenem-nonsusceptible *Enterobacteriaceae* isolates collected in Taiwan. Siméon et al. [12] reported the use of cefiderocol to treat a case of prosthetic joint infection due to extensively drug-resistant *Enterobacter hormaechei*. Karruli et al. [13] identified the use of central venous catheters and length of hospitalization as risk factors of multidrug-resistant infections after heart transplants in a single-center study.

In conclusion, the genomic epidemiology of multidrug-resistant organisms, the analysis of molecular determinants of antimicrobial resistance, the identification of innovative diagnostic and therapeutic approaches are important to prevent and control antimicrobial resistance.
